# A Comprehensive Review of the Nano-Abrasives Key Parameters Influencing the Performance in Chemical Mechanical Polishing

**DOI:** 10.3390/nano15171366

**Published:** 2025-09-04

**Authors:** Houda Bellahsene, Saad Sene, Gautier Félix, Joulia Larionova, Marc Ferrari, Yannick Guari

**Affiliations:** 1 Institut Charles Gerhardt Montpellier (ICGM), Centre National de la Recherche Scientifique (CNRS), École Nationale Supérieure de Chimie de Montpellier (ENSCM), University of Montpellier, 34293 Montpellier, France; houda.bellahsene@etu.umontpellier.fr (H.B.); saad.sene@umontpellier.fr (S.S.); gautier.felix@umontpellier.fr (G.F.); joulia.larionova@umontpellier.fr (J.L.); 2 Laboratoire d’Astrophysique de Marseille (LAM), Centre National de la Recherche Scientifique (CNRS), Centre National d’Etudes Spatiales (CNES), Aix Marseille University, 13013 Marseille, France; marc.ferrari@osupytheas.fr

**Keywords:** chemical mechanical polishing, abrasive, nanoparticles

## Abstract

Chemical Mechanical Polishing (CMP) is a critical process in many industries where achieving superior surface quality through controlled material removal rates by using nano-abrasives is essential. This review examines key parameters of abrasives at the nanoscale, such as size, shape, aspect ratio, hardness, and surface modifications, through inorganic doping or organic molecule grafting and their influence on CMP performance. By analyzing recent studies, we explore how these parameters affect the tribological and chemical interactions during CMP and link these effects to the fundamental polishing mechanisms. Highlighting emerging trends, this work offers a roadmap for designing next-generation nano-abrasives that boost removal efficiency, enhance surface finish, and ensure process stability. Ultimately, controlling abrasive properties at the nanoscale is vital for advancing CMP technology toward more efficient, consistent, and high-quality results.

## 1. Introduction

**Figure 1:**
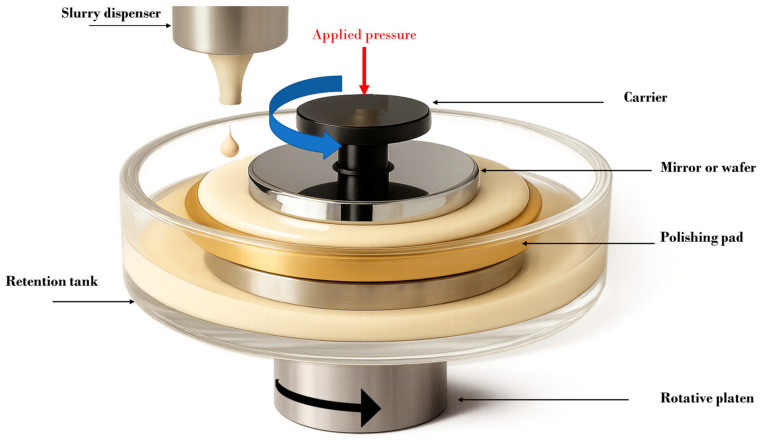
Simplified schematic representation of a CMP setup.

A wide range of fields including artificial intelligence (AI), smart manufacturing, automotive electronics, the Internet of Things (IoT), 5G communications, exoplanet detection, and synchrotron beamline instrumentation depend on critical technological components with precisely engineered surface properties. Depending on the nature of the surface to be polished and the specific application, different planarization techniques can be considered. These include Ion Beam Figuring (IBF), Magnetorheological Finishing (MRF), Fluid Jet Polishing (FJP), Elastic Emission Machining (EEM), plasma-assisted polishing, and, finally, Chemical Mechanical Polishing (CMP) [1]. CMP, which remains a widely used and versatile method across multiple advanced technology sectors, offers excellent control over surface topography and roughness, while requiring careful management of potential wafer deformation or damage induced by abrasive or chemical reactions; making it particularly well-suited when ultra-smooth surfaces with ultra-low damage are required [2,3,4,5]. Notably, surface roughness (*R_a_*) values, which is the arithmetic average of the absolute values of surface height deviations measured from the mean line over a 2D profile, can be reduced to below 1 nanometer by using this technique [6]. CMP was initially introduced and commercialized by IBM in the 1980s for silicon wafer planarization in microelectronics manufacturing [7]. The main components of a CMP system include a polishing pad, typically made of a porous polymer, such as polyurethane, which ensures uniform distribution of the slurry, a mixture of abrasive particles and chemicals, and a carrier head that holds the surface to be polished. During operation, the pad and/or the carrier head rotate while the surface is pressed against the pad. The combined mechanical abrasion from the pad and chemical action from the slurry remove material in a controlled way, resulting in a highly flat and smooth surface (Figure 1).

The final surface quality, namely roughness, flatness and subsurface damage, depends on several key process parameters, such as applied pressure, rotation speed, slurry composition, slurry flow rate or stepwise addition, pad type, polishing time, and temperature, among others. The influence of each parameter can vary significantly depending on the complexity of the system and the materials involved. The slurry itself represents a complex, multiparametric aspect of the CMP process. Its composition naturally depends on the material to be polished, but it typically includes five components: an abrasive, a dispersant, an oxidant, a pH regulator, and a surfactant. Each component has a specific function in the process, as detailed in Table 1.

The slurry plays a crucial role in the CMP process, being involved in both the chemical and the mechanical dynamics. Among its various components, the abrasive plays a key role, as it can contribute to both the chemical modification of the surface and its mechanical removal. Since abrasive-related parameters directly impact the efficiency of the CMP process, significant research has been devoted to their optimization. The performance of abrasives is typically evaluated based on their contribution to the Material Removal Rate (*MRR*) and/or the surface roughness (*R_a_*) of the polished substrate.

The CMP results can be analyzed from a mechanical perspective based on the indentation-based or the surface area-based mechanisms [8,9,10,11]. The polish rates corresponding to the former (Equation (1)) and the latter (Equation (2)), respectively, can be given as follows:

*MRR* α *V* α *C*_0_^−1/3^*d*^4/3^ (1)

*MRR* α *A* α *C*_0_^1/3^*d*^−1/3^ (2)

Here, *MRR* represents the polish rate, while *A* and *V* correspond to the contact area and the indentation volume, respectively. *C_0_* denotes the abrasive concentration, and *d* refers to the diameter of the abrasive particles. It is commonly accepted that the *R_a_* of the polished substrate is primarily determined by the indentation depth of abrasive particles into the substrate surface which is influenced by the mechanical properties of the abrasive particles, such as their hardness (*H*) and Young’s modulus (*E_a_*). Additionally, both mechanisms are governed by the size of the abrasive particles and, to a certain extent, by their shape factor, which may further modulate their mechanical interaction with the substrate.

**Figure 2:**
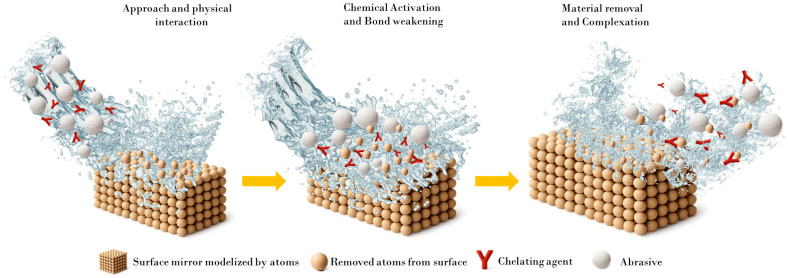
Simplified schematic representation of the different phases in CMP process.

The chemical aspect of the CMP process is primarily influenced by the surface chemistry and composition of both the substrate and the abrasive, but also by the overall chemical environment of the slurry, including its various additives. The different stages of the chemically assisted material removal mechanism [12,13] can be described in four successive phases (Figure 2):

(i) approach and physical interaction between the abrasive and the substrate, where both mechanical and chemical aspects are involved. The abrasive must come into close proximity with the substrate surface, a process driven by mechanical forces (e.g., downforce, pad-abrasive-substrate contact dynamics), but modulated by chemical affinities such as surface charge interactions and colloidal stability;
(ii) chemical activation and bond weakening, wherein the abrasive interacts with the substrate at the atomic scale, forming transient bonds that chemically modify the surface and reduce the bond strength within the substrate lattice. Note that this step is highly sensitive to the surface chemistry and reactivity of the abrasive, as well as to the local pH, redox conditions, and presence of catalytic species in the slurry;
(iii) material detachment, where sufficient mechanical energy, which supplied by the abrasive motion and contact forces, is required to overcome the reduced bond strength and enable the removal of substrate atoms. This stage is predominantly mechanical but occurs efficiently only when preceded by adequate chemical activation;
(iv) complexation and stabilization of the removed species, typically through chelating agents present in the slurry that bind to the liberated atoms or reaction intermediates, preventing re-adsorption or particle agglomeration.

The abrasive plays a central role particularly in the first two stages. While its bulk chemical composition remains critical, it is the surface physicochemical properties that most strongly influence its effectiveness. These include surface charge, hydrophilic/hydrophobic balance, surface atom electronegativity [14], and the nature, density, and accessibility of surface functional groups [15,16,17]. These characteristics determine not only the abrasive’s chemical reactivity but also its colloidal behavior and mechanical interaction potential with the substrate.

The field of CMP has been the subject of several recent reviews, with particular emphasis on the diverse nature of abrasive materials. They have addressed various categories, including silica [18] and ceria-based abrasives [19], abrasives incorporating rare-earth elements [20], or composite abrasives [21,22]. Moreover, environmental considerations associated with the use of abrasives in CMP process have also been addressed [23]. In this overview, we will focus on the various chemical parameters of abrasives, particularly at the nanoscale, that can specifically influence either the mechanical or chemical components of the CMP process. Although it remains challenging to fully decorrelate their effects on these two components, recent studies have primarily focused on changes in size, shape factor, or hardness of the nanoabrasive, which we have already highlighted as playing a dominant role in the mechanical aspects. On the other hand, modifications to the surface chemistry of nanoabrasives, whether through inorganic doping or functionalization with organic groups, are expected to predominantly affect the chemical component of CMP. By examining both physical and chemical characteristics, this review aims to identify general trends in how these distinct parameters contribute to enhancing polishing efficiency and achieving surface qualities tailored to the requirements of different application areas introduced earlier.

## 2. Physical Properties of Nanoabrasives

### 2.1. Size

The impact of nano-abrasive particle size on CMP performance has been extensively studied, especially in applications involving the use of silica nanoparticles for planarizing metallic or dielectric surfaces. Understanding this effect is critical for optimizing CMP processes and achieving high precision in surface finishing [24]. When the main removal mechanism relies on the indentation-based mechanical action of abrasive particles pressing into the surface, for a fixed concentration *C*_0_, *MRR* value increases with the particle diameter *d* raised to the power of 4/3, as shown in Equation (1). This reflects how larger particles exert greater mechanical force during polishing. Alternatively, when the predominant mechanism involves chemical reactions enhanced by the contact surface area of abrasive particles, the *MRR* is expected to be proportional to the total surface area of abrasives in contact with the wafer. In this case, the removal rate depends on the total abrasive surface area *A*, which is a function of both the particle size *d* and the volume concentration *C*_0_, as given Equation (2). This can also be expressed in terms of the number concentration of abrasive particles, *N*, as follows:

*N* α *C*_0_/d^3^

and thus

*MRR* α *A* α *Nd*^2^ α *C*_0_/d (3)

**Figure 3:**
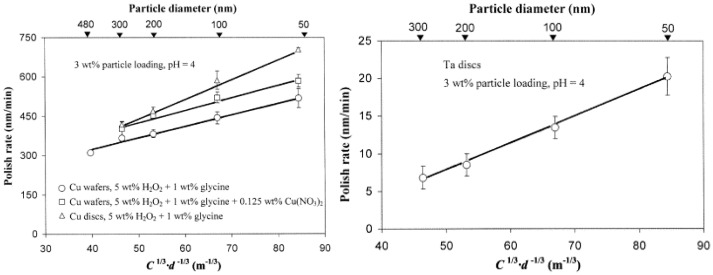
*MRR* for Cu (**left**) and Ta (**right**) polishing using silica nanoabrasives with different sizes plotted according to the contact area model. Adapted from Ref. [25] with permission from Elsevier.

This relationship highlights that smaller particles, present in greater numbers for a fixed volume concentration, offer a larger total surface area, thereby enhancing surface area-driven chemical reactions. This tendency has been demonstrated experimentally using silica nanoparticles with sizes ranging from 50 to 300 nm for the polishing of copper and tantalum (Figure 3). It has been observed that the *MRR* of copper wafers, as well as copper and tantalum discs, increases as the size of the silica nano-abrasives decreases. Furthermore, it has been established that the polishing rate remains independent of particle size when slurries with the same total surface area of dispersed abrasives are used [25].

Moreover, in the context of CMP of glass using a colloidal silica-based slurry with particle sizes ranging from 40 to 120 nm, the observed dependence of the *MRR* on particle size has suggested the coexistence of two operative mechanisms within the system under study. At a fixed slurry concentration, the dominant mechanism appears to shift with particle size: in the range of 40 to 60 nm, an indentation driven mechanism prevails, leading to an increase in *MRR* as particle size increases. Conversely, as particle size increases beyond 60 nm up to 120 nm, a surface-area-driven mechanism becomes dominant, resulting in a decrease in *MRR* with increasing particle size [9]. A similar trend has been observed in the CMP of silicon wafers using silica nanoparticles ranging from 10 to 140 nm, where the MRR increases with particle size up to 80 nm, then decreases as the particles become larger [26].

The size effect plays also a critical role in determining the final surface condition, particularly influencing surface roughness (*R_a_*). For the CMP of aluminum alloy substrates with NiP coating using silica nanoparticles ranging from 15 to 160 nm [27], the *MRR* increases steadily with particle size, in contrast to previously reported cases [9,26]. Nevertheless, the surface roughness of the polished surfaces follows the same trend: as the average diameter of the SiO_2_ particles increases from 15 to 160 nm, *R_a_* decreases first, reaching a minimum value around 30 nm, and then gradually increases. In other words, achieving high surface quality depends on selecting an appropriate abrasive particle size, with the optimal size of silica particles under the given conditions being approximately 30 nm. Since material removal is typically accompanied by friction, smaller abrasive particles, which are more numerous at a fixed volume concentration, lead to an increased total contact area. As a result, frictional forces tend to increase as particle diameter decreases. To better understand the nature and origin of friction in CMP, the coefficient of friction (*COF*) was measured during the polishing of fused silica by using silica particles with diameters ranging from 25 to 500 nm. Interestingly, although the average *COF* values for silica particles with diameters of 100 and 500 nm were comparable, a statistically significant increase was observed when using 25 nm particles [28]. However, it is difficult to fully decorrelate the effect of particle size from other contributing factors in CMP. In particular, the stability of nanoparticles can be strongly influenced by their size, with smaller nanoparticles often exhibiting a higher tendency to aggregate. This behavior can compromise polishing performance and may require the addition of stabilizing agents to the slurry formulation. One of the effective approaches involves using amines that carry a positive charge at the working pH, which helps enhance nanoparticle dispersion. This strategy has been shown to yield a favorable compromise between achieving a high *MRR* and a lower *R_a_*, particularly when using silica nanoparticles in the 15–20 nm range, compared to those between 50 and 70 nm [29]. Finally, it is important to consider that CMP is inherently a dynamic process, and during closed-loop polishing, the particle size may evolve over time. In a study of silicon wafer polishing using SiO_2_ nanoparticles with initial diameters of 20 and 55 nm, a slight increase in nanoparticle size of 2–3% was observed after 30 min of polishing [30]. In this case, the *MRR* associated with the nanoparticles of 20 nm was lower than that observed with the 55 nm particles, suggesting that the predominant material removal mechanism is the indentation-based mechanism. This interpretation is further supported by the observed increase in *MRR* over time for the 20 nm particles, consistent with a gradual increase in particle size enhancing the indentation effect.

The effect of abrasive nanoparticles’ size on CMP performance is nuanced and highly dependent on the dominant material removal mechanism. For the indentation-based mechanism, larger particles tend to increase the *MRR* due to their greater mechanical impact. In contrast, surface-area-driven mechanism favors smaller particles, which provides a higher total contact area and supports more uniform, chemically assisted material removal. One may consider that the use of sub-micrometric particles, and particularly nanoparticles, sizes smaller than 100 nm, would be expected to promote a surface-area-driven mechanism, while it has been demonstrated that under certain conditions both mechanisms may coexist [9,26]. In addition to *MRR* efficiency, the size of abrasive particles impacts the surface quality. It is well established now that indentation-based mechanisms are more likely to cause surface defects such as scratches or micro-indentations, resulting from localized mechanical interactions between larger abrasives and the substrate. Consequently, these processes often lead to increased roughness. By contrast, surface-area-based mechanisms, which involve a larger number of smaller particles such as nanoparticles with lower indentation potential, tend to yield smoother surfaces. Therefore, the use of particles at the nanoscale may offer a meaningful *MRR* while maintaining, or even improving, surface quality (*R_a_*), thus meeting the increasingly stringent requirements of advanced CMP applications. However, despite its significance, particle size alone does not fully account for the observed variations, requiring the consideration of additional factors.

### 2.2. Shape Factor

Over the past decade, numerous studies have investigated the use of non-spherical silica nanoparticles for the CMP of various surfaces, most often in comparison with spherical nanoparticles of similar size. Several shape typologies have been explored, including peanut-like, heart-shaped, ellipsoidal, and nanorod forms. In the latter cases, an important geometric parameter referred as the *aspect ratio*, a dimensionless quantity that describes the proportional relationship between its length, *l*, and width, *w*, can be defined as:

*Aspect Ratio* = *l*/*w* (4)

The evaluation of performance in CMP using non-spherical nanoparticles, compared to spherical counterparts of equivalent size, typically results in a noticeable increase in *MRR* for various types of surfaces, such as SiO_2_, Al_2_O_3_, or ZrO_2_ [31,32,33,34,35]. Chen et al. [36] analyzed mechanical effect by establishing three models for *MRR* that are the abrasive model, the adhesive model, and the erosive model which is considered to be the least contribution to the overall material removal rate. The abrasive and adhesive models can be expressed as:

*MRR_abrasive_* = *aδU* (5)

*MRR_adhesive_* = (*k_a_π*/2)*a*d*U*  (6)

where *MRR_abrasive_* is the *MRR* from abrasive wear, *MRR_adhesive_* is the *MRR* from adhesive wear, *a* is the actual contact radius, *δ* is the penetration depth, *U* is the rotating speed of polishing pad, *ka* is the coefficient of adhesive wear, and *d* is the atomic diameter of wafer. The contact area of spherical silica nanoparticles is confined to a single point due to their regular geometry, whereas the contact area of irregular silica nanoparticles may involve a small surface. Consequently, the contact area between the nanoparticle and the surface is increased, potentially accelerating the chemical reaction occurring at the interface. More importantly, when other conditions remain constant, the *MRR* is expected to increase as the actual contact radius expands, as described by Equations (5) and (6). Furthermore, spherical and irregular nanoparticles can behave differently upon contact with the surface. Spherical silica nanoparticles are likely to engage in rolling motion during the chemical mechanical polishing process. In contrast, due to their irregular shape, silica nanoparticles tend to favor sliding over rolling, which enhances the mechanical interaction with the surface and contributes to a higher material removal rate. This explanation is further supported by a study measuring both the *MRR* and the friction coefficients, which we have previously discussed in Section 2.1. The study demonstrated that the increase in MRR for non-spherical nanoparticles, compared to spherical ones of comparable size correlates with the measured friction coefficients [37]. In fact, the non-spherical colloidal silica slurry exhibited a higher *COF* during CMP, indicating greater frictional forces between the pad, particles, and wafer surface throughout the polishing process. As the abrasive particles interact with the polishing pad during CMP, mechanical actions, such as rolling and sliding, occur at the pad-particle-wafer interface. Higher sliding motion of abrasives results in greater friction forces compared to those produced by rolling abrasives. For instance, the elongated shape of non-spherical colloidal silica particles resists rolling, causing them to spend more time in sliding motion, effectively removing material from the wafer surface. Consequently, non-spherical colloidal silica particles exhibit higher *COF* and friction force with the surface being polished, leading to a higher *MRR* compared to spherical particles.

**Figure 4:**
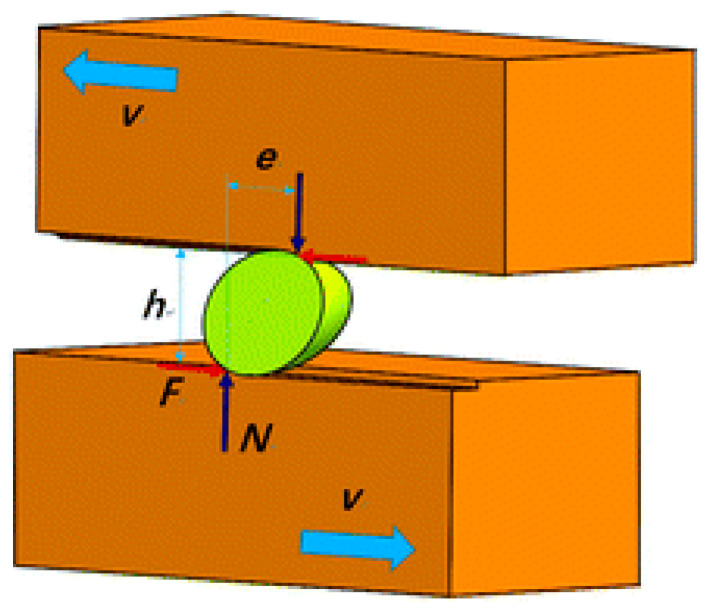
Cross-sectional schematic illustration of the three-body abrasion process for an ellipsoid particle, where *N* represents the normal load, *F* denotes the friction coefficient, and *v* is the sliding velocity. Reproduced from Ref. [38] with permission from the Royal Society of Chemistry.

Two studies utilizing dynamic Molecular Dynamic simulations support this hypothesis and provide a deeper understanding of this phenomenon [38,39]. They specifically assessed the role of a geometric parameter, *e*/*h*, in relation to the hardness of the material being abraded, the normal load, the frictional load, and the shape of the particle. In the expression of the geometric parameter, *e* represents an equivalent arm for the resistant moment, and *h* is the driving moment (Figure 4). From the geometric parameters, a criterion for the particle movement pattern can be proposed. When a particle slides, the condition is:

*e*/*h* ≥ *μ_s_*  (7)

where *μ_s_* is the friction coefficient for a sliding particle. Similarly, if a particle rolls, the condition becomes:

*e*/*h* < *μ_r_* (8)

where *μ_r_* is the friction coefficient for a rolling particle. Thus, when the friction coefficient is smaller than or equal to *e*/*h*, the particle slides; the particle rolls when the friction coefficient is larger than *e*/*h*.

Depending on the system considered, such as monocrystalline diamond ellipsoidal particles sandwiched between monocrystalline copper or crystalline silicon workpieces, the transition between rolling and sliding occurs at *axial ratios* of 0.83 and 0.46, with sliding predominating below these values. The *axial ratio* defines the particle shape, which, in the case of these studies, corresponds to the inverse of the *aspect ratio* (Equation (4)) and can be defined as follows:

*Axial Ratio* = 1/*Aspect Ratio* = *w*/*l* (9)

where *l* represents the length and *w* is the width. Thus, a complete sphere will have an *axial ratio* of 1 and a flattened ellipsoid an *axial ratio* of 0.5. This transition can be predicted by applying the criterion for particle movement patterns, especially when elastic recovery is considered. At the nanoscale, elastic recovery plays a crucial role and cannot be neglected as it often is at the macroscale. It significantly influences particle behavior in three-body abrasion, where rolling leads to greater defect depth, groove depth, and dislocation length compared to sliding. As a result, although sliding is associated with a higher average friction coefficient and a higher *MRR*, it should tend to yield better final surface quality due to reduced subsurface damage.

**Figure 5:**
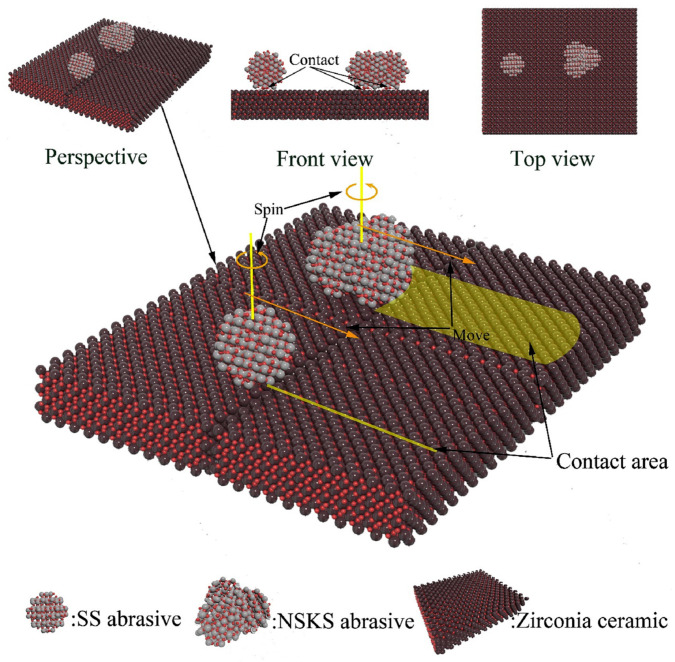
Schematic illustration of the contact wear model of spherical and heart-shaped silica nanoparticles for CMP of ZrO_2_. SS refers to Spherical Silica and NSKS to Non-Spherical silica. Adapted from Ref. [35] with permission from Elsevier.

Such an effect on the final surface quality was experimentally demonstrated in a study comparing peanut-like and heart-shaped nanoparticles with spherical nanoparticles of comparable size [35]. In this case, assuming all other parameters remain constant, the final *S_a_* value obtained after polishing was 2.4 nm when using non-spherical nanoparticles, compared to 3.2 nm for spherical nanoparticles, starting from an initial *S_a_* of 6.7 nm. This corresponds to a 64% reduction in surface roughness with non-spherical nanoparticles, versus 52% with spherical ones. It is worth noting that *S_a_* is the three-dimensional counterpart of *R_a_*, and thus represents the arithmetic mean of the absolute height deviations from a reference plane over a 3D surface area. For spherical nanoparticles, the particles first rotate in solution and then come into point contact with the wafer, followed by a certain degree of translation. The resulting interaction is characterized by a one-dimensional line contact as depicted Figure 5. In contrast, for heart-shaped nanoparticles, the particles rotate around their own axis in solution due to multi-point contact with the wafer, which transforms the interaction into a one-dimensional coil contact. This is followed by translational motion, resulting in a two-dimensional surface contact. Consequently, the contact area generated by heart-shaped nanoparticles is significantly larger than that produced by spherical nanoparticles. The measured friction coefficients and *MRR* are correspondingly higher for the non-spherical nanoparticles than for the spherical ones. Notably, this study introduces the possible nanoparticle spin motion on the surface that could be particularly relevant for nanoparticles with a high *aspect ratio*, such as elongated particles, for which the contact surface becomes even more significant.

A systematic study was conducted to investigate the effect of *aspect ratio* on the CMP of Cadmium Zinc Telluride wafers using SiO_2_ particles with sizes in the hundreds of nanometers [40]. These particles exhibited a morphology resembling that of a cube for an *aspect ratio* close to 1, and increasingly elongated rectangular parallelepipeds for *aspect ratios* ranging from 1.35 to 3.5. A comparison was also made with spherical particles of equivalent size. In this case, the best results in terms of final surface quality were obtained using the cubic nanoparticles (*aspect ratio* close to 1). However, it is noteworthy that these nanoparticles are mesoporous, which introduces an additional parameter, as porosity significantly affects both the hardness (*H*) and the Young’s modulus (*E_a_*) of the particles: two parameters that are particularly crucial for CMP, as it will be discussed in the following section.

The modification of nanoparticle shape has a clear impact on CMP performance, influencing both the *MRR* and the final surface quality. This effect is primarily attributed to a transition in particle motion; from rolling, typically observed for nanoparticles with *aspect ratios* close to 1, to sliding for more elongated particles. Sliding motion induces higher *COF*, which is directly correlated with increased *MRR*. Moreover, despite the higher *COF* and *MRR*, sliding appears to result in improved final surface quality due to reduced subsurface damage. These observations may support that, under the specific experimental conditions fixed, a predominant surface-area-driven mechanism may be considered as a unifying explanation for the enhanced performance of non-spherical nanoparticles in CMP. In other words, the use of smaller nanoparticles is generally identified as a means of achieving high-quality final surface finishes, albeit at the expense of material removal efficiency. The use of nanoparticles with an *aspect ratio* greater than one could therefore be considered a suitable compromise, offering both an acceptable *MRR* and excellent surface quality, in line with the specific requirements of various targeted applications.

### 2.3. Hardness

#### 2.3.1. Porosity

In addition to the example discussed in the previous section concerning the influence of the shape of mesoporous SiO_2_ particles on the CMP of silicon wafers [40], few studies have focused exclusively on the effect of porosity, particularly for large particles with the size of the several hundred nanometers. For them, mesoporosity is induced through the use of a template, such as sodium dodecyl sulfate for Al_2_O_3_ particles or hexadecyltrimethylammonium bromide for SiO_2_ particles, whose post synthetic removal creates the porous structures. This strategy allows for creation of pore volumes in the range of 0.35–0.46 cm^3^/g for Al_2_O_3_ and 0.69–0.83 cm^3^/g for SiO_2_, values significantly higher than those obtained for the synthesis of the same particles without the addition of a template agent. The reported works on the use of mesoporous Al_2_O_3_ particles for the CMP of NiP-plated aluminum alloy [41,42], or mesoporous SiO_2_ particles for the CMP of silicon wafers [43] demonstrated that the use of porous particles led to a notable improvement in surface roughness (*Ra*) compared to non-porous or less porous counterparts of similar size. Specifically, mesoporous Al_2_O_3_ particles resulted in a 5–18% reduction in *Ra* compared to non-porous Al_2_O_3_, and mesoporous SiO_2_ particles achieved a 41% improvement compared to non-porous SiO_2_.

This effect has been attributed to the fact that porous particles exhibit lower hardness (*H*) and Young’s modulus (*E_a_*) compared to equivalent-sized particles with limited porosity, which leads to a decrease in contacting stress and indentation depth between the abrasives and substrates, ultimately helping to reduce the finished surface roughness. In the case of CMP of NiP-plated aluminum alloy with Al_2_O_3_ particles, no significant effect was observed on the *MRR*. In contrast, for the CMP of a silica wafer with mesoporous SiO_2_ particles, the *MRR* was found to be twice as high as that of particles of the same size but with lower porosity. The authors explain this effect by noting that mesoporous SiO_2_ particles, with their high pore volumes and specific surface areas (up to 1400 m^2^/g), are able to adsorb more active chemical components from the slurry, thereby enhancing the chemical reactivity in the local contact region between the abrasives and substrates. As a result, this improves the *MRR*, is in line with the contact-area-based mechanism.

A more extensive set of experimental data on the effect of porosity on CMP, particularly for the use of nanoparticles, would provide a clearer understanding of these various effects on CMP performance.

#### 2.3.2. Core–Shell

**Figure 6:**
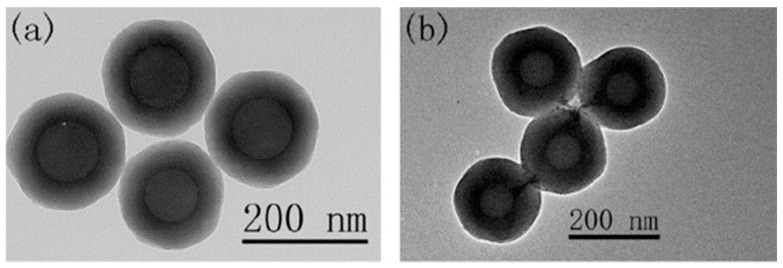
TEM images of representative synthesized polystyrene-SiO_2_ core–shell nanoparticles with (**a**) 100 nm polystyrene core and 46 nm SiO_2_ shell (**b**) 75 nm polystyrene core and 58 nm SiO_2_ shell. Adapted from Ref. [44] with permission from Elsevier.

Among the wide range of core–shell architectures that can be envisioned, most reported studies have focused on core–shell systems featuring SiO_2_ or CeO_2_ as the shell material. In these configurations, the core typically consists of polystyrene, Al_2_O_3_, Fe_3_O_4_, or CeO_2_ covered by SiO_2_ shells, and conversely, SiO_2_ cores are often used when the shell is composed of CeO_2_. Considering polystyrene particles coated with SiO_2_, L. Zhang et al. focused on investigation of core size and shell thickness impact on the nanoparticle’s performance for the CMP of a copper surface [44]. They developed core–shell particles in which the polystyrene core size was varied from 55 to 150 nm, while the SiO_2_ shell thickness ranged from 15 to 64 nm (Figure 6). The performance of nanoparticles with a 75 nm polystyrene core and a 58 nm SiO_2_ shell, resulting in a total diameter of 191 nm, was then compared to that of 119 nm pure SiO_2_ particles. Starting from an initial *S_a_* of 8.47 nm, the use of SiO_2_ particles reduced the *S_a_* to 4.27 nm, corresponding to a 50% reduction in surface roughness. In comparison, the use of polystyrene–SiO_2_ core–shell particles further decreased the *S_a_* to 0.56 nm, representing a 93% reduction. The *MRR* was slightly lower for the SiO_2_ nanoparticles, at 42 nm/min, compared to 45 nm/min for the polystyrene- SiO_2_ core–shell nanoparticles.

A more recent study on polystyrene- SiO_2_ core–shell nanoparticles, featuring a 300 nm polystyrene core and a SiO_2_ shell thickness varying from 35 to 200 nm, focused on the influence of shell thickness on CMP performance during the polishing of a silicon wafer [45]. The observed effects were further analyzed using small deformation contact theory, supported by measurements of the elastic moduli of the polystyrene–SiO_2_ core–shell particles, to elucidate the relationship between shell thickness, particle deformation, and polishing efficiency. The progressive increase in the *MRR* with decreasing shell thickness was successfully modeled using the measured elastic moduli, along with the calculated contact radius (*a*) and indentation depth (*δ*) (see Equation (5)). Similarly, the surface roughness was found to decrease progressively as the shell thickness and consequently the elastic modulus decreased. Thus, the use of a polystyrene core leads to an increase in *MRR* and a decrease in *S_a_*, with both effects becoming more pronounced as the SiO_2_ shell thickness decreases. This behavior can be attributed to an optimal balance between a low elastic modulus and the presence of a reactive SiO_2_ surface, which is essential for effective polishing, as polystyrene alone has no chemical effect on the surfaces. A similar effect has also been reported for polystyrene-CeO_2_ core–shell particles, which results in improved *R_a_* values compared to pure CeO_2_ nanoparticles, although the latter are 10 times smaller in size, in the context of polishing reaction-bonded silicon carbide (RB-SiC) [46]. However, this improvement in surface quality comes at the expense of a reduced *MRR*, which can, however, be compensated by leveraging the chemical effect through UV irradiation in photocatalytic assisted CMP (PCMP), and using CeO_2_-TiO_2_ instead of pure CeO_2_.

It has also been shown that the presence of a SiO_2_ shell can reduce the hardness of the abrasive itself, thereby having a positive effect on the *R_a_*. This was demonstrated in the case of Al_2_O_3_ particles (~300 nm) coated with a silica layer used for the CMP of NiP-plated aluminum alloy [47]. In this case, *R_a_* decreases with increasing SiO_2_ content in the shell (up to ca. 25%). However, this improvement is accompanied by a significant reduction in *MRR* (up to ca. 20%). This trade-off is attributed to the difference in chemical reactivity between Al_2_O_3_ and SiO_2_ with respect to the polished surface. It is also worth noting that replacing Al_2_O_3_ surface with SiO_2_ alters the dispersibility of the particles due to the more negative zeta potential observed under CMP conditions with a SiO_2_ shell. This enhanced surface charge contributes as an additional factor to the performance differences observed during polishing.

Finally, for core-SiO_2_ shell structures, one example involves the growth of a SiO_2_ layer around Fe_3_O_4_ nanoparticles, not with the aim of modifying the hardness or the surface state of the nanoabrasive, but rather to impart magnetic properties, enabling its recovery and potential reuse in the CMP of BK7 optical glass [48].

**Figure 7:**
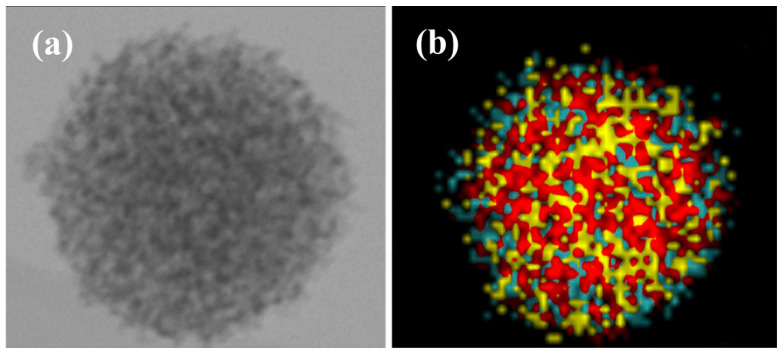
(**a**) STEM image and (**b**) overlap of EDX elemental mapping of Si (red), Ce (grey-blue) and Sm (yellow). Reproduced from Ref. [52] with permission from Springer Nature.

Other reported examples of nanoscale core–shell structures or assimilated designed for use in CMP consist in SiO_2_-CeO_2_ type architectures. Thus, CeO_2_ nanoparticles have been formed on the surface of SiO_2_ nanoparticles, based on the idea that uniform colloidal CeO_2_ is difficult to produce, particularly in the large amounts required for CMP studies, making this strategy a practical and effective alternative for such applications [49]. It is worth noting that the authors do not classify the obtained nanoheterostructures as core–shell due to the incomplete coating, but rather describe them as a smooth silica surface, studded with ceria spikes. When those SiO_2_-CeO_2_ nanoparticles were used for the CMP of grown silicon oxide wafers, a remarkable increase in *MRR* is observed compared to their individual components, namely silica or ceria nanoparticles, or physical mixtures of the two. While *MRR* values range from 11 to 18 nm/min for the individual components and from 19 to 54 nm/min for physical mixtures of different ratio, SiO_2_-CeO_2_ nanoheterostructures demonstrated *MRR* between 91 and 115 nm/min. This corresponds to an increase of up to 945% compared to the lowest individual component and up to 113% compared to the highest-performing mixture. Polishing rates for the latter are essentially independent of their concentration, pH, or size. This late finding led the authors to propose that in this system, both the indentation-based mechanism (Equation (1)) and the surface-area-based mechanism (Equation (2)) are present, compensating for the potential effect of size, as *MRR* should decrease with increasing size for the surface-area-based mechanism, while it should increase for the indentation-based mechanism. In line with these results, other studies focused on SiO_2_ nanoparticles coated with CeO_2_, this time with a complete coating, leading to core–shell nanoparticles for their use in CMP of grown silicon oxide wafers. These studies also show a significant increase in *MRR* when using SiO_2_-CeO_2_ core–shell nanoparticles compared to pure SiO_2_ nanoparticles. Under the most favorable conditions, the use of SiO_2_-CeO_2_ nanoparticles of 260 ± 20 nm achieves an *MRR* of 454.6 nm/min, which represents a 420% increase compared to 87.5 nm/min for SiO_2_ nanoparticles of 160 ± 10 nm [50]. A similarly significant increase in *MRR* was observed in another study for the use of SiO_2_-CeO_2_ core–shell nanoparticles compared to SiO_2_ nanoparticles, with an 811% increase, along with good stability of *MRR* for the core–shell nanoparticles as a function of pH [51]. Finally, an interesting example concerns the construction of core–shell structures, combining a mesoporous SiO_2_ core at the nanoscale, coated with CeO_2_ nanoparticles, either doped or undoped with samarium (Figure 7) [52]. In this case, non-agglomerated mesoporous SiO_2_ nanoparticles with an initial size of 83 ± 9 nm were coated with CeO_2_ nanoparticles, resulting in a final size of 97 ± 12 nm, or 103 ± 16 nm when the CeO_2_ was doped with samarium. This homogeneous coating with doped or undoped CeO_2_ nanoparticles leads to the formation of core–shell nanostructures. These last two types of nanoparticles, along with commercial CeO_2_ nanoparticles ranging from 100 to 200 nm in size, were considered for the CMP of grown silicon oxide wafers, which had an initial *S_a_* of 0.93 nm. While the commercial particles result in an *MRR* of 72 nm/min with a *S_a_* of 0.41 nm, the core–shell nanoparticles achieve *MRR* values of 106 nm/min and 151 nm/min, with *S_a_* values of 0.18 nm and 0.16 nm when the ceria nanoparticles are undoped or doped with samarium, respectively. These values correspond to surface roughness reductions of 56% for the commercial particles, compared to 81% and 83% for the undoped and Sm-doped core–shell nanoparticles, respectively. The core–shell nanoparticles used produce similar *S_a_* values, resulting from similar mechanical properties, i.e., lower *H* and *E_a_* compared to the commercial ceria particles. Thus, the mesoporous silica core imparts an increased elastic response to the core–shell nanoabrasives, leading to a reduction in *S_a_* but also to a decrease in mechanical damages such as scratches or etching pits [53]. On the other hand, the difference in *MRR* is highly significant, with a 50% increase when the ceria nanoparticles constituting the shell are doped with samarium. This significant effect and its origin are described in more detail in the following section.

Therefore, the use of core–shell nanoparticles in CMP offers a strategy for combining complementary properties within a single nanoabrasive. This approach enables the integration of a softer component while preserving a reactive surface. Such configurations allow for the modulation of the nanoabrasive’s hardness and Young’s modulus, which can improve final surface roughness while maintaining *MRR* efficiency. However, unlike the above discussed approach, where hardness can be adjusted through nanoparticle porosity without significantly altering the surface state, the use of core–shell nanoparticles to achieve similar objectives introduces more complex modifications. The growth of a shell affects the surface state, which in turn influences chemical reactivity, surface charge, and nanoparticle stability depending on pH. These modifications are intricate and must be carefully optimized to maximize the benefits of the individual components in these specialized architectures. Nevertheless, this approach may also be useful for reasons other than modifying *H* or *E_a_*. As demonstrated, it provides the advantage of generating a significant quantity of a specific nanoparticle type, that may be difficult to achieve, on the surface of another particle that is easily synthesized in large quantities and serves as a support. The growth of a soft, reactive shell can also facilitate the incorporation of magnetic properties throughout the core, solely for the purpose of simplifying the separation and reuse of the nanoabrasive. The number of possible combinations is therefore substantial, offering significant prospects, although the synthetic complexity of this approach can pose a significant limiting factor for its practical use.

## 3. Surface Chemical Properties of Nanoabrasives

### 3.1. Surface Doping of Chemical Elements

In the previous section, we discussed a case study involving core–shell nanoparticles wherein the shell composed of CeO_2_ was doped with samarium to enhance the *MRR* [52]. Such a strategy to enhance the efficiency of CeO_2_ nanoparticles has also been reported in other studies over the past few years. Indeed, the enhancement in the *MRR* observed upon doping CeO_2_ with lanthanide ions can be attributed to the defect chemistry induced by the substitution of Ce^4+^ with trivalent lanthanide cations (Ln^3+^). This substitution process introduces charge imbalance, which is compensated by the formation of oxygen vacancies and a partial reduction of Ce^4+^ to Ce^3+^ [54]. The doping mechanism can be described by the following chemical reactions using Kröger-Vink notation, where the substitution of Ce^4+^ by Ln^3+^ results in the formation of oxygen vacancies (Vö) that can be represented as follows:

Ln_2_O_3_ → 2Ln^′^_Ce_ + 3O_O_^x^ + Vö (10)

The partial reduction of Ce^4+^ to Ce^3+^ is closely associated with the creation of oxygen vacancies. Indeed, as shown in the following equations, the creation of an oxygen vacancy is accompanied by the release of free electrons, which are subsequently captured by Ce^4+^, leading to their reduction to Ce^3+^:

O_O_^x^ → 1/2O_2(g)_ + Vö+ 2é (11)

Ce^4+^ + é → Ce^3+^ (12)

where Ln^′^_Ce_ refers to the substitution of Ce^4+^ by Ln^3+^ (effective negative charge), O_O_^×^ is an oxygen on its usual site (neutral), and Vö is an oxygen vacancy with an effective positive charge (missing two electrons).

**Figure 8:**
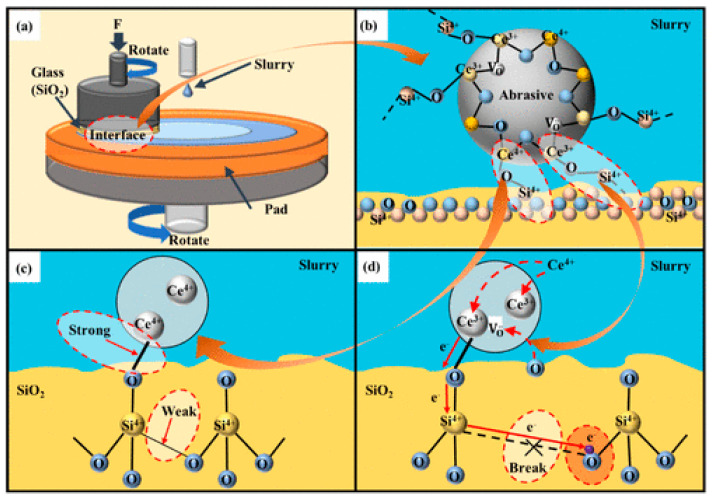
Schematic diagrams of (**a**) CMP model, (**b**) removal model of CeO_2_ abrasives, and (**c**,**d**) reaction model between Ce^4+^/Ce^3+^ and the SiO_2_ substrate. Adapted with permission from Ma, J. et al., 2023 [56]. Copyright 2021 American Chemical Society.

To explain the enhancement of the *MRR* with increasing Ce^3+^ content, it is essential to consider that interfacial electron transfer occurs within ceria [55]. Accordingly, the interaction mechanism between Ce^3+^ and the SiO_2_ substrate may involve the transfer of free electrons. They are transferred from Ce^3+^ to the SiO_2_ surface upon the formation of Ce–O–Si interfacial bonds during CMP, which weakens, or even disrupts, the stable Si–O covalent bonds (Figure 8d). The presence of Ce^3+^ in the abrasive phase thus significantly enhances Si–O bond cleavage efficiency, leading to an increased *MRR* [56].

Thus, doping CeO_2_ nanoparticles with various lanthanide ions leads to variable, yet often significant, improvements in the *MRR* compared to their undoped counterparts. For instance, in the CMP of thermally grown SiO_2_ wafers, increases in the SiO_2_ removal rate of 50.0, 20.9, 29.6 and 4.3% have been reported for Sm/CeO_2_ [52], La/CeO_2_, Nd/CeO_2_ and Yb/CeO_2_ [54], respectively. In the case of K9 glass, La/CeO_2_ achieved an increase of 70.5% in the removal rate [56]. In all cases, the increase in *MRR* does not come at the expense of the final surface quality. It should be noted that Yb/CeO_2_ exhibits the highest Ce^3+^ content compared to La/CeO_2_ and Nd/CeO_2_ [52], yet the enhancement in silica polishing rate is not as significant (only 4.3%). Therefore, the chemical interaction between Ce^3+^ and the dielectric surface is just one of the factors influencing CMP, and other factors, such as electrostatic interactions, contact angle, size, morphology of the abrasive particles, and more generally, the mechanical aspects, also have to be considered [57].

Hence, surface doping of nanoparticles represents an effective strategy to tailor their chemical reactivity with respect to the substrate being polished. This strategy has also been investigated using SiO_2_ nanoparticles approximately ~80 nm in diameter, doped with various ions at different concentrations, with the aim of introducing additional reactive pathways to enhance the efficiency of CMP. Specifically, doping with Nd^3+^ (0.25–1 wt%) resulted in the formation of surface species, such as Nd_2_SiO_7_ and Nd(OH)_3_; Cu^2+^ led to the formation of Cu(OH)_2_ (1.0–2.0 wt%), Co^2+^ yielded Co(OH)_2_ (0.5–1.5 wt%), Mg^2+^ produced Mg(OH)_2_ (0.5–1.5 wt%), and Ce^4+^ gave rise to CeO_2_ (0.5–1.5 wt%) [58,59,60,61,62]. These doped nanoparticles were subsequently employed in the polishing of sapphire (α-Al_2_O_3_), and the underlying mechanisms were investigated using various analytical techniques to identify the species formed during the abrasion process. Significant enhancements in both *MRR* and surface quality were achieved across all cases, with maximum improvements relative to undoped SiO_2_ nanoparticles reaching up to 85% increase in *MRR* and up to 59% reduction in *R_a_*, depending on the optimal doping level for each ion. From a mechanical standpoint, the authors suggest that the presence of additional compounds reduces the hardness (*H*) of the nanoparticles, which may negatively affect the mechanical contribution to the polishing process beyond certain thresholds. Notably, the highest *MRR* values are observed at these optimal doping levels; for example, 0.75 wt% for Nd^3+^ doping and 0.5 wt% for CeO_2_. From a chemical standpoint the reactions between the surface of undoped silica nanoparticles and the sapphire surface can be represented as follows:

Al_2_O_3_ + SiO_2_ ⇋ Al_2_SiO_5_ (13)

Al_2_O_3_ + H_2_O + 2SiO_2_ ⇋ Al_2_Si_2_O_7_·H_2_O (14)

When the surface is doped with species, such as Nd(OH)_3_ and Nd_2_Si_2_O_7_, Cu(OH)_2_, Co(OH)_2_, Mg(OH)_2_, or CeO_2_, they can introduce additional reactivity. This enhanced chemical interaction with the sapphire surface can be described by the following reactions:

3Al_2_O_3_ + 2Nd(OH)_3_ + 3SiO_2_ ⇋ Nd_2_Al_2_Si_3_O_12_ + 4AlOOH + H_2_O (15)

Al_2_O_3_ + Nd_2_Si_2_O_7_ + SiO_2_ ⇋ Nd_2_Al_2_Si_3_O_12_ (16)

Al_2_O_3_ + Cu(OH)_2_ ⇋ Al_2_CuO_4_ + H_2_O (17)

Al_2_O_3_ + Co(OH)_2_ ⇋ Al_2_CoO + H_2_O (18)

Al_2_O_3_ + 2Mg(OH)_2_ + 8SiO_2_ + Na_2_O ⇋ 2NaAlMg(Si_4_0_10_)(OH) (19)

Al_2_O_3_ + CeO_2_ + SiO_2_ ⇋ Al_x_SiCeO_y_ (20)

Beyond introducing an additional reactive functionality through doping, other strategies have focused on imparting catalytic functionality to the nanoparticle. This is notably the case in a study focusing on the doping of ~80 nm SiO_2_ nanoparticles with Ag^+^ in the range of 0.5–1.5 wt% for the polishing of sapphire surfaces [63]. As can be seen in the following reactions, the presence of the dopant induces additional reactivity towards the substrate, with the key difference compared to the previous cases that the species responsible for this activity is regenerated during the process, rendering it catalytic.

2Ag^+^ + 2OH^−^ ⇋ Ag_2_O + H_2_O (21)

3SiO_2_ + 3/2Al_2_O_3_ + NO_3_^−^ + 20 Ag_2_O +3/2H_2_O ⇋ Al_3_N_5_O_3_Si_3_ + 4AgO + 5OH^−^ (22)

2AgO + H_2_O +2e^−^ ⇋ Ag_2_O + 2OH^−^ (23)

This additional reactivity leads to a progressive enhancement in both *MRR* and surface quality with increasing doping levels, compared to undoped SiO_2_ nanoparticles, reaching up to a 123% increase in *MRR* and a 59% reduction in *R_a_* at the highest doping level studied (1.5 wt%). Other relevant examples in the field of catalysis involve photocatalysis, where UV irradiation during the polishing process enables the generation of reactive oxygen species (ROS) to enhance CMP performance. Such an approach has been the subject of studies focusing on the use of polystyrene-CeO_2_/TiO_2_ core–shell structures [46], as well as mesoporous SiO_2_ nanoparticles onto whose surfaces CeO_2_ and CuO nanoparticles were formed [64]. These studies therefore combine two factors that can enhance CMP performance: mechanical aspects discussed previously, through the modification of hardness and Young’s modulus relative to the presence of a soft core, and chemical aspects, through the generation of reactive oxygen species (ROS) by modifying the surface. Nonetheless, the mechanisms by which ROS are generated may differ between the two nanoabrasives used. In the first case, the production of ·OH radicals result from the formation of photoinduced holes (h^+^), which subsequently react with OH^−^ or H_2_O according to the following reactions:

TiO_2_/CeO_2_ +hν → h^+^ + e^−^ (24)

h^+^ + OH^−^ → ·OH (25)

h^+^ + H_2_O → ·OH (26)

In the second case, ROS generation may also proceed via the same mechanism through CeO_2_, but can additionally result from a heterogeneous Fenton-like reaction, due to the presence of copper on the nanoparticle surface and H_2_O_2_ in the slurry according to the following reactions:

Cu^2+^ + H_2_O_2_ → Cu^+^ + ·O_2_^−^ + 2H^+^ (27)

Cu^+^ + H_2_O_2_ → Cu^2+^ + ·OH + OH^−^ (28)

**Figure 9:**
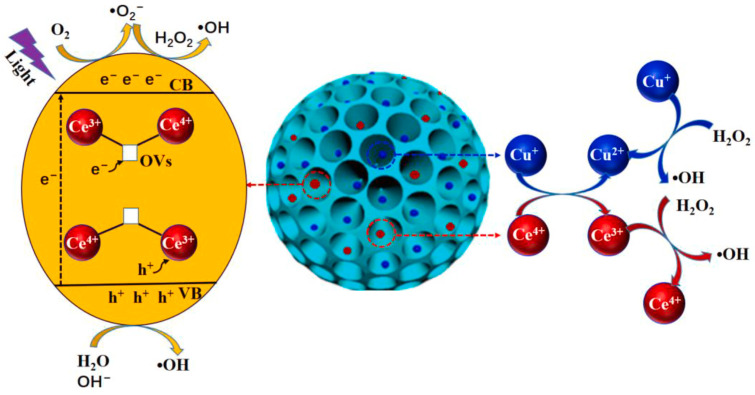
Schematic representation of the possible mechanisms of ROS generation for mesoporous SiO_2_ nanoparticles functionalized with surface-deposited CeO_2_ and CuO nanoparticles. Adapted from Ref. [64] with permission from Elsevier.

In this case, a cooperative effect can also be envisioned due to the presence of both CeO_2_ and CuO, involving electron transfer between the Cu^+^/Cu^2+^ and Ce^3+^/Ce^4+^ redox couples (Figure 9).

It should be noted, however, that in the case of polystyrene-CeO_2_/TiO_2_ core–shell nanoparticles, the photocatalytic contribution cannot be considered as resulting from doping, since it originates from the entire shell, which contains both components in significant proportions. Similarly, in the second study, it is difficult to assess whether the term “doping” is appropriate, as the exact amounts of CeO_2_/CuO in the resulting nanocomposites have not been reported. That said, in both cases, the photocatalytic component has a significant impact on *MRR* and *R_a_*. For instance, in the case of polystyrene-CeO_2_/TiO_2_ core–shell nanoparticles used for polishing RB-SiC surfaces, improvements of 441% increase in *MRR* and 86% decrease of *R_a_* were observed under UV-assisted CMP (PCMP) conditions compared to standard CMP. Similarly, for mesoporous SiO_2_ nanoparticles decorated with CeO_2_ and CuO and used in the polishing of thermally grown SiO_2_ on Si wafers, improvements of 21% in *MRR* and 15% reduction in *R_a_* were reported under PCMP conditions compared to CMP.

Thus, surface doping represents a valuable strategy for tailoring the chemical reactivity of nanoparticle surfaces to enhance CMP performance, which can arise from an increased proportion of highly reactive species, such as Ce^3+^ generated via Ln^3+^ doping of CeO_2_. Moreover, this effect can be induced by the introduction of additional chemical reaction pathways, as demonstrated by the surface doping of SiO_2_ nanoparticles with ions, such as Nd^3+^, Cu^2+^, Co^2+^, Mg^2+^, Ce^4+^, or Ag^+^. In this latter case, the dopants may also impart catalytic functionality, whereby the active species is regenerated during the reaction, sustaining its effect throughout the polishing process. Similarly, photocatalytic approaches, although not strictly categorized as surface doping in the reported studies, have shown that PCMP can significantly enhance both *MRR* and *R_a_*. These various approaches, including doping and photocatalysis, collectively underscore the broader potential of modifying nanoparticle surfaces to optimize CMP efficiency and improve both *MRR* and surface quality.

### 3.2. Surface Functionalization with Organic Groups

Similarly, several studies have focused on the surface functionalization of nanoabrasives with reactive organic groups, aiming to achieve the enhancement of the CMP process. Table 2 provides an overview of various organic functional groups grafted onto the surface of nanoabrasives, as well as the different types of nanoabrasives and substrate surfaces considered in the context of polishing.

Surface functionalization of nanoabrasives with functional organic moieties can exert multiparametric influences on CMP. By modifying the surface characteristics of the abrasives, such functionalization may alter their dispersion behavior and the stability of the slurry, facilitate the approach and physical interaction between the abrasive and the substrate, and/or enhance chemical activation and bond weakening at the contact interface. These effects are themselves strongly dependent on the density, nature, and chemical reactivity of the grafted functionalities. The influence of grafting density, in particular was highlighted in a study investigating the functionalization of SiO_2_ and Al_2_O_3_ nanoparticles with chemically inert fluorinated chains for the polishing of copper surfaces in supercritical CO_2_ (scCO_2_) [65]. In this context, the primary objective of fluorinated functionalization is to enhance the CO_2_-philicity of the nano-abrasives, thereby improving their dispersion in the medium. However, when SiO_2_ nanoparticles are functionalized at a grafting density of 1.4 chains/nm^2^, the surface hydroxyl groups exhibit little to no chemical interaction with the copper substrate due to shielding by the C_8_F_17_ tails. As a result, the *MRR* is comparable to that observed in the absence of nano-abrasives. This is consistent with observations in scCO_2_ (~40 °C, 214 bar), where these fluorinated moieties promote strong particle-solvent interactions, indicating that the tails adopt an extended conformation in this good solvent regime [73]. Conversely, at a lower grafting density of 1.1 chains/nm^2^, a greater number of intact oxide and hydroxyl groups remain accessible, enabling stronger interactions with copper surfaces and resulting in a 33% increase in the *MRR*. These findings underscore the necessity of balancing surface functionalization: while improving nanoparticle dispersion and slurry stability, it must also preserve sufficient surface reactivity, particularly when using chemically inert grafts, to ensure a positive impact on CMP performance. Surface functionalization with a grafting agent to improve abrasive dispersion stability has also been investigated in two studies focusing on the polishing of soda lime glass using Al_2_O_3_ particles. In these cases, the grafting molecule used was 3-(Trimethoxysilyl)propyl methacrylate, a coupling agent capable of initiating free-radical graft polymerization of methacrylic acid (MAA). This approach ultimately leads to the formation of core–shell Al_2_O_3_-poly(methyl methacrylate) (PMMA) particles (see Section 2.3.2) [66,67]. While the dispersion of the abrasive is significantly improved through this surface modification, the efficiency of the CMP process in terms of *MRR* is reduced by approximately 50% due to surface passivation. Nevertheless, the formation of the core–shell Al_2_O_3_-PMMA structure leads to a reduction in both the hardness and Young’s modulus of the abrasive particles. This, combined with the enhanced dispersibility, contributes to an improved final surface quality, with the *Ra* decreasing by 16–39%, depending on the polishing conditions.

**Figure 10:**
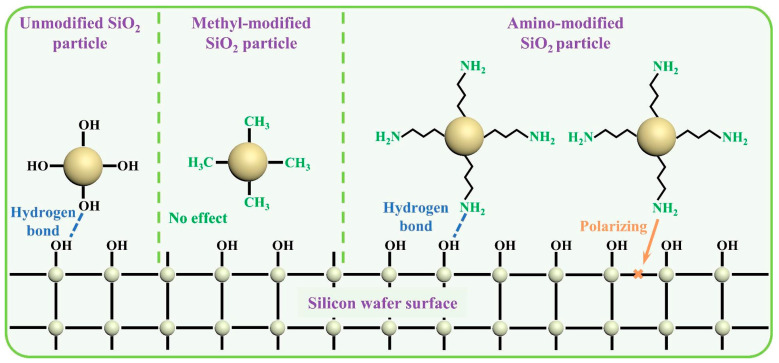
Schematic representation of the interactions between unmodified SiO_2_ nanoparticles and those functionalized with methyl groups or APTES with the Si wafer. Adapted from Ref. [71] with permission from Elsevier.

In contrast to the previously described cases, it is also possible to introduce ligands that do not passivate the abrasive surface, but confer additional chemical reactivity. This is exemplified by the functionalization of 17–20 nm SiO_2_ nanoparticles with N-[3-(Trimethoxysilyl)propyl]isothiouronium chloride (TSIC) for the polishing of substrates, such as polycrystalline silicon (poly-Si), SiO_2_, or Si_3_N_4_ [68]. In this case, the authors propose that the amine functionality present on the surface of the nano-abrasive can bind to the poly-Si surface and polarize Si–Si bonds due to its inductive effect. This hypothesis is supported by parallels with studies in which primary amines (arginine, lysine, ornithine, or guanidine carbonate) are directly added to the slurry [74,75]. As previously discussed, grafting density is one of the key parameters to consider when functionalizing the surface of nano-abrasives. If the grafted moiety is chemically inert with respect to the substrate, an increase in grafting density should lead to a decrease *MRR* due to surface passivation. Conversely, if the grafted group is chemically reactive toward the substrate as proposed in the present case, increasing the grafting density should have a positive effect on the *MRR* by enhancing surface interactions and promoting bond destabilization. This is indeed the case in the present study, where the grafting density was controlled by varying the amount of TSIC added during the surface functionalization of SiO_2_ nanoparticles (17–20 nm in diameter). At pH 10, the polishing rate of SiO_2_ substrates increased from 40 nm/min without TSIC to 70 nm/min with 3% TSIC and up to 100 nm/min with 5% TSIC (which corresponds to ca. 3 TSIC/nm^2^), reflecting improvements of +75% and +150%, respectively. These results support the idea that the use of chemically reactive ligands can enhance polishing performance, likely by strengthening interfacial interactions and facilitating bond destabilization at the substrate surface. Several studies have investigated the functionalization of CeO_2_ or SiO_2_ nanoparticles with 3-aminopropyltriethoxysilane (APTES), in order to improve the CMP performance on various substrates. An initial study using APTES focused on the functionalization of CeO_2_ nanoparticles for the polishing of soda-lime glass [69]. While unmodified ceria nanoparticles fully precipitated after one day, APTES-functionalized particles remained well dispersed in water even after 14 days. This improved colloidal stability was accompanied by a narrower particle size distribution, as observed by dynamic light scattering: the median diameter decreased from 447.6 nm for unmodified ceria to 158.7 nm for functionalized particles. Although some aggregation persisted, the surface modification significantly enhanced dispersibility. The suppression of ceria particle agglomeration led to a 31% reduction in the *MRR* and a 43% decrease in surface roughness (*R_a_*) when the CMP process was done at pH 4. All subsequent studies on APTES functionalization focused on SiO_2_ nanoparticles, with an initial investigation evaluating the performance of functionalized nanoparticles at different pH levels (4.0, 7.3, and 9.9) for the polishing of fused silica glass [70]. The *MRR* for a conventional slurry was 0.375 µm/h, whereas APTES-functionalized nanoparticles achieved *MRRs* of 1.5 µm/h in acidic conditions, 0.25 µm/h at neutral pH, and 0.5 µm/h in basic media, corresponding to *MRR* increases of 300%, 177%, and 355%, respectively. In addition, surface roughness (*R_a_*) was reduced by 40%, 21%, and 28% under acidic, neutral, and basic conditions, respectively. Thus, the effectiveness of the CMP process, both in terms of *MRR* and *R_a_*, as a function of pH, follows a clear trend: the highest *MRR* (1.5 µm/h) and the greatest reduction in *R_a_* (40%) are observed in acidic conditions, where APTES is predominantly in its protonated form (NH_3_^+^). Basic conditions show a *MRR* of 0.5 µm/h and a 28% reduction in *R_a_*, with APTES in its neutral (NH_2_) form. Neutral conditions exhibit the lowest *MRR* (0.25 µm/h) and a moderate reduction in *R_a_* (21%), with APTES also in its neutral form. Finally, two studies investigated the functionalization of SiO_2_ nanoparticles with APTES for silicon wafer polishing under basic conditions (pH > 10), and compared the results with those obtained using trimethoxymethylsilane. One study showed that, relative to unmodified nanoparticles, APTES functionalization resulted in a 24% increase in *MRR*, while trimethoxymethylsilane led to an 18% decrease [71]. For Si wafer polishing, OH^−^ ions strongly polarize surface Si–Si bonds, enabling water and dissolved oxygen (or added oxidant in the slurry) to attack them, which leads to bond breakage and/or the formation of Si–O–Si suboxide structures. These suboxides are weakly bound to the underlying polysilicon layer and are easily removed during polishing. Repeated cycles of suboxide formation and removal result in continuous material removal. Consequently, as the OH^−^ concentration increases with pH, the silicon removal rate also increases. Silica abrasives further enhance suboxide removal through mechanical abrasion, leading to higher *MRRs* compared to those obtained using only pH-adjusted water. In this context, the use of surface-modified SiO_2_ particles showed that amino-functionalized particles resulted in even higher *MRRs*, while methyl-functionalized ones led to reduced *MRRs*. This can be attributed to the role of amino groups, whose nitrogen atoms promote the reaction between water and Si–Si bonds more effectively than OH^−^ ions. Additionally, amino groups enhance adhesion to the wafer surface through hydrogen bonding with surface Si–OH groups, improving polishing efficiency. Conversely, methyl groups do not possess properties like amino groups or OH^−^ ions, and the substitution of Si-OH on the particle surface with a methyl group weakens the hydrogen bonding between the particles and the wafer surface, leading to a lower *MRR* (Figure 10).

However, another study, employing similar approach, reports contradictory results, showing a lower *MRR* for APTES-functionalized nanoparticles compared to both unmodified nanoparticles and those functionalized with methyl groups. This can be attributed to the formation of a polymeric layer on the nanoparticle surface during APTES functionalization, which conceals the amine groups, rendering them unavailable to exert the positive effect on *MRR*, as previously discussed [72]. Regarding the improvement of the surface state, a similar discrepancy in the results is observed, not only for the effect of APTES-functionalized nanoparticles, which can again be explained by an altered surface state between the two studies, but also for methyl-functionalized nanoparticles, with one study showing an increase in *R_a_* and the other a decrease.

Thus, surface functionalization of nanoparticles with grafted organic groups allows for the modulation of nanoabrasive effectiveness in CMP by influencing dispersion state, stability, and chemical reactivity towards the polished surface. The discussed examples demonstrate that chemically inert groups can passivate the surface, often leading to lower *MRR*, although sometimes the improved dispersion can compensate for this effect. In contrast, reactive chemical groups, particularly primary amines, which have been studied notably on glass or Si wafer surfaces, show a highly positive effect, enhancing *MRR* and often reducing *R_a_* as well. Therefore, functionalization with organic grafted groups appears to be an interesting parameter for fine-tuning the CMP process, although this modulation remains complex, as evidenced by sometimes contradictory results in the literature for closely related systems.

## 4. Conclusions

The CMP process is a complex, multiparametric approach that inherently combines both tribological and chemical components. Its performance is highly dependent on polishing conditions depending on the complexity of the system under study. The slurry itself constitutes a particularly intricate aspect of the CMP process, with a highly variable composition that may include up to five key constituents. One of these components, the abrasive, can be tailored in terms of both physical properties (e.g., particle size, shape factor, hardness) and chemical characteristics (e.g., surface functionalization with inorganic or organic entities). Each modification introduced to the abrasive may simultaneously influence multiple aspects of the CMP process, thereby complicating the interpretation of its specific impact on polishing efficiency. Moreover, the widespread focus on optimizing CMP conditions for each specific system—typically, in terms of material removal rate (*MRR*) and surface roughness (*Ra*)—often comes at the expense of establishing standard polishing protocols. While such standardized conditions may not yield optimal performance for any given system, they would greatly facilitate comparison across studies by fixing a set of polishing parameters and isolating the influence of a single variable coming from the abrasive.

Nonetheless, when focusing more specifically on nanoscale abrasives (typically <100 nm, though often extending to a few hundred nanometers in many studies within the field), the current state of knowledge and technological advancement already allows for the identification of relevant parameters, which can be systematically adjusted, with certain promising directions having clearly emerged. In the context of this review, and to facilitate the discussion of the various parameters involved, we have chosen to present them according to their physical properties, which predominantly influence the tribological aspects of the process, and their chemical modifications, which primarily affect the interfacial chemical reactions during CMP. Nevertheless, as we have emphasized on several occasions, this distinction remains somewhat arbitrary, as the modification of a given parameter can simultaneously impact both the tribological and chemical aspects of the CMP.

Accordingly, a first set of variables that can be investigated for nanoabrasives pertains to their physical characteristics, such as particle size and shape factor, as well as textural features that may influence their hardness and Young’s modulus. Although abrasives at the nanoscale are expected to promote surface-area-driven mechanisms, both surface-area-based and indentation-based mechanisms can coexist under certain conditions, with surface-area-driven processes not always being dominant. Nanoparticles are particularly advantageous for applications requiring precise planarization with minimal surface damage. Indentation-based mechanisms tend to cause surface defects, such as scratches and micro-indentations, increasing roughness, whereas surface-area-driven mechanisms, involving smaller particles, tend to yield smoother surfaces. Therefore, nanoscale abrasives can provide an acceptable, though not optimal, *MRR* but may offer a significant gain in surface quality. Another relevant parameter that has been the focus of increasing attention is the particle shape, and more specifically, the *aspect ratio*. This effect is attributed to a transition in particle motion, from rolling for nearly spherical nanoparticles to sliding for more elongated ones. Sliding increases the *COF*, which is linked to higher *MRR*. In addition, sliding also improves surface quality by reducing subsurface damage. These observations suggest that a surface-area-driven mechanism can explain the enhanced performance of non-spherical nanoparticles in CMP. Thus, nanoparticles with an *aspect ratio* greater than one strike a balance, offering both higher *MRR* and excellent surface quality. The modification of *H* and *Ea*, whether through the introduction of porosity, the design of core–shell structures, or a combination of both, has also been studied, particularly in recent years. These modifications influence not only the tribological behavior of the nanoabrasives, but also their chemical reactivity. For instance, introducing porosity can enhance local reactivity by allowing reactive species to be stored within the pores, thereby increasing overall polishing efficiency. Similarly, the formation of core–shell structures enables tuning of tribological properties, but also has a direct impact on surface reactivity, not merely due to the structural modification itself, but because of the resulting change in surface chemistry. This dual effect makes it difficult to extract a general trend, as it is highly specific to each system under study. Nonetheless, it remains a highly interesting parameter, as it can be strategically exploited by simultaneously controlling both the mechanical properties of the nanoheterostructure and the chemical nature of its surface.

Beyond modifying the physical properties of nanoabrasives, it is also possible to focus on altering their surface reactivity. One approach involves introducing an inorganic dopant via metal ions, which can enhance CMP performance. For instance, Ln^3+^ surface doping of CeO_2_ generates more reactive Ce^3+^ towards the surface to polish. Moreover, doping nanoparticles’ surface with metal ions can introduce additional chemical reaction pathways. In some cases, dopants may also provide catalytic functionality, regenerating the active species during the reaction and sustaining its effect throughout the polishing process. Similarly, photocatalytic approaches, i.e., photo-assisted CMP, may lead to significant improvements in both *MRR* and surface quality. Another approach involves functionalizing the surface of nanoabrasives with organic groups, which can modulate their reactivity. This strategy offers the potential to fine-tune the chemical interactions between the abrasive and the polished surface, ultimately influencing the effectiveness of the CMP process. At the same time, functionalization with organic groups allows for the modulation of dispersion, stability, and reactivity towards the polished surface. Organic groups can alter the surface reactivity, either enhancing or diminishing efficiency depending on the specific interaction with the substrate and the number of grafted groups present. For example, chemically inert groups can passivate the surface, often resulting in a lower *MRR*, though this effect can sometimes be compensated by improved dispersion. In contrast, incorporating reactive groups, such as primary amines, particularly on surfaces like glass or Si wafers, has been shown to significantly enhance *MRR* while often reducing surface roughness. Therefore, surface functionalization either with inorganic dopants or organic groups on various proportions provides a powerful yet complex tool for optimizing the CMP process.

CMP plays a critical role in various industrial processes where the achievement of high-quality surface finishes is essential. The advancements made so far have enabled the development of surfaces with exceptional quality, yet there is a growing need to push beyond these achievements due to the increasingly demanding requirements of current applications, as exemplified for precision optics in areas such as exoplanet detection and synchrotron beamlines, where even higher precision is essential for enhanced performance and accuracy. In this context, the race for minimal yet significant gains has made the nanoabrasive an object of increasing research interest. This area is poised to become a significant research field in which nanomaterial chemists will play a crucial role in the coming years. To yield meaningful insights into the phenomena under investigation, it may be necessary to establish standardized polishing conditions. Such standards, if widely adopted, would enable direct comparisons of the effects of different nanoabrasives, facilitating the identification of clear trends in the observed outcomes. Additionally, with the contribution of artificial intelligence applied to a large body of comparable data, it will be possible to trace and analyze these trends more effectively, thereby not only advancing the optimization of the desired surface states but also helping to identify key areas for further study, which will enhance our fundamental comprehension of the overall process. Finally, in an important application context, the development of efficient and simple nanoabrasives will be essential to enable scalability and keep costs within reasonable bounds. This consideration should be integrated early into the synthetic approaches envisioned. Furthermore, attention should also be given to waste management, particularly considering the large quantities of slurries used in CMP. It is crucial to address sustainability issues, and from the perspective of nanoabrasives, this could involve the design of self-repairing or catalytic nanoabrasives to extend their lifespan and enable their reuse, while maintaining a stable process over time.

## Figures and Tables

**Table 1:** Components of the slurry and their functions.

Slurry Component	Function
Abrasive	The abrasive provides the mechanical and chemical actions needed to remove material from the surface. These fine particles, commonly silica (SiO_2_), alumina (Al_2_O_3_), or ceria (CeO_2_), interact with the wafer under pressure and motion, contributing to surface planarization.
Dispersant	The dispersant helps maintain a stable and uniform distribution of abrasive particles in the slurry. It prevents agglomeration and sedimentation, ensuring consistent polishing performance and avoiding defects like scratching or uneven removal (e.g., polyacrylic acid).
Oxidant	The oxidant in the slurry promotes chemical reactions that modify the surface of the material being polished. This typically results in the formation of a thin, soft oxide layer that is easier to remove by the abrasive particles, improving the efficiency of material removal and enabling selective polishing (e.g., H_2_O_2_).
pH regulator	The pH regulator controls and maintains the acidity or alkalinity of the slurry. By adjusting the pH, it helps optimize the chemical reactions between the oxidant and the material being polished, ensuring consistent material removal rates and preventing unwanted side reactions or damage to the surface (e.g., HNO_3_ or KOH).
Surfactant	The surfactant in CMP slurry helps reduce surface tension, improving the wetting properties of the slurry. This ensures better contact between the abrasive particles, the pad, and the surface being polished. It also aids in the dispersion of abrasive particles, preventing agglomeration and ensuring uniform polishing across the surface (e.g., polyethylene glycol, sodium lauryl sulfate).

**Table 2:** Organic functional groups grafted onto the surface of nanoabrasives.

Nanoabrasive	Surface Substrate	Grafted Organic Group	References
SiO_2_	Cu	-O(CH_3_)_2_(CH_2_)_2_C_8_F_17_	[65]
Al_2_O_3_	Cu	-O_3_Si(CH_2_)_2_C_8_F_17_	[65]
Al_2_O_3_	Soda Lime Glass	-O_3_Si(CH_2_)_3_OC(=O)C(=CH_2_)CH_3_	[66,67]
SiO_2_	PolySi or SiO_2_ or Si_3_N_4_	[-O_3_Si(CH_2_)_3_S^+^=C(NH_2_)_2_]Cl^−^	[68]
CeO_2_	Soda Lime Glass	-O_3_Si(CH_2_)_2_NH_2_	[69]
SiO_2_	Fused silica Glass	-O_3_Si(CH_2_)_2_NH_2_	[70]
SiO_2_	Si	-O_3_Si(CH_2_)_2_NH_2_ or -O_3_SiCH_3_	[71]
SiO_2_	Si	-O_3_Si(CH_2_)_2_NH_2_ or -O_3_SiCH_3_	[72]

## Data Availability

No new data were created or analyzed in this study.

## Abbreviations

The following abbreviations are used in this manuscript:

AI	Artificial Intelligence
APTES	3-AminoPropylTriEthoxySilane
CMP	Chemical Mechanical Polishing
COF	Coefficient Of Friction
Ea	Young’s Modulus
EEM	Elastic Emission Machining
FJP	Fluid Jet Polishing
H	Hardness
IBF	Ion Beam Figuring
IoT	Internet of Things
MAA	MethAcrylic Acid
MRF	Magnetorheological Finishing
MRR	Material Removal Rate
PCMP	Photocatalytic-assisted Chemical Mechanical Polishing
PMMA	Poly(Methyl MethAcrylate)
Ra	Arithmetic Mean Surface Roughness
RB-SiC	Reaction-Bonded Silicon Carbide
ROS	Reactive Oxygen Species
Sa	Areal Arithmetic Mean Surface Roughness
scCO_2_	supercritical CO_2_
TSIC	N-[3-(Trimethoxysilyl)propyl]isothiouronium chloride
UV	UltraViolet
